# Identifying Candidates for Effective Utilization of Stored Autologous PBSCs in Salvage Transplantation for Multiple Myeloma: Who Benefits Most?

**DOI:** 10.3390/hematolrep16030046

**Published:** 2024-07-12

**Authors:** Amany R. Keruakous, Laura Walker, Molly Denlinger, Mohammad A. H. Mian, Danielle Bradshaw, Vamsi K. Kota, Anand P. Jillella

**Affiliations:** Division of Hematology and Oncology, Georgia Cancer Center, Augusta University, Augusta, GA 30912, USA; lwalker4@augusta.edu (L.W.); mdenlinger@augusta.edu (M.D.); mmian@augusta.edu (M.A.H.M.); dabradshaw@augusta.edu (D.B.); vkota@augusta.edu (V.K.K.); ajillella@augusta.edu (A.P.J.)

**Keywords:** stem cell collection, autologous stem cell transplantation, cost utilization, multiple myeloma

## Abstract

**Background/Objectives:** High-dose chemotherapy (HD-CHT) followed by autologous stem cell transplantation (ASCT) remains the gold standard for eligible multiple myeloma (MM) patients, even amidst evolving therapeutic options. Clinical trials have demonstrated ASCT’s efficacy in MM, including its potential as salvage therapy after prolonged remission. Peripheral blood stem cells (PBSCs) are now the primary source of hematopoietic stem cells for ASCT. Collecting additional PBSCs post-initial myeloablative conditioning is challenging, leading many centers to adopt the practice of collecting and storing excess PBSCs during initial therapy to support tandem transplants or salvage treatments. The use of salvage ASCT may diminish in the face of novel, highly effective treatments like bispecific antibodies and cellular therapies for relapsed/refractory MM (RRMM). Despite available stored PBSC grafts, salvage ASCTs are underutilized due to various factors, including declining performance status and therapy-related comorbidities. A cost utilization analysis from 2013 revealed that roughly 70% of patients had unused PBSC products in prolonged cryopreservation, costing a significant portion of total ASCT expenses. The average cost for collecting, cryopreserving, and storing PBSCs exceeded $20,000 per person, with more than $6700 spent on unused PBSCs for a second ASCT. A more recent analysis from 2016 underscored the declining need for salvage ASCT, with less than 10% of patients using stored PBSC grafts over a decade. **Methods:** To address the dilemma of whether backup stem cells remain necessary for myeloma patients, the study investigated strategies to reduce the financial burden of PBSC collection, processing, and storage. It evaluated MM patients undergoing frontline ASCT from January 2012 to June 2022, excluding those with planned tandem transplants and those who had a single ASCT with no stored cells. **Discussion:** Among the 240 patients studied, the median age at PBSC collection was 61. Notably, only 7% underwent salvage ASCT, with nearly 90% of salvage ASCT recipients being ≤ 61 years old at the time of initial ASCT. The study revealed a decreasing trend in salvage ASCT use with increasing age, suggesting that PBSC collection for a single transplant among elderly patients (>60 years old) could be a cost-effective alternative. Most transplant centers aimed to collect 10 × 10^6^ CD34 + cells/kg, with patients over 65 often requiring multiple collection days. Shifting towards single-transplant collections among the elderly could reduce costs and resource requirements. Additionally, the study recommended implementing strategies for excess PBSC disposal or repurposing on the collection day to avoid additional storage costs. In summary, the decreasing utilization of salvage ASCT in MM, alongside financial considerations, underscores the need for revised stem cell collection policies. **Conclusions:** The study advocates considering single-transplant PBSC collections for elderly patients and efficient management of excess PBSCs to optimize resource utilization.

## 1. Introduction

Multiple myeloma (MM) is a hematological malignancy characterized by the proliferation of malignant plasma cells within the bone marrow [1]. High-dose chemotherapy (HD-CHT) followed by ASCT has been a standard and effective treatment approach for eligible MM patients for several decades. However, the optimal strategy for stem cell collection and storage has evolved over time, shaped by clinical outcomes, financial considerations, and advances in MM therapeutics [2]. This study will examine key factors contributing to the utilization of stored peripheral blood stem cells (PBSCs) for a salvage ASCT, shedding light on its impact on patient care and resource allocation.

### 1.1. Historical Perspective

Early approaches to stem cell collection primarily utilized bone marrow as the source of hematopoietic stem cells for ASCT in MM. This method required invasive bone marrow aspirations and was associated with limited yields. Consequently, it was gradually supplanted by the use of peripheral blood stem cells (PBSCs), which could be collected via leukapheresis, offering higher stem cell yields and a less invasive procedure [3].

### 1.2. Shift to PBSCs

The shift from bone marrow to PBSCs as the primary source of stem cells for ASCT in MM is well-documented in the literature [4]. PBSCs are now the preferred choice due to their ease of collection and superior stem cell yield. Several clinical trials and observational studies have demonstrated that PBSC transplantation results in faster hematopoietic recovery, shorter hospital stays, and potentially improved overall survival when compared to bone marrow transplantation [5].

### 1.3. The Role of Salvage ASCT

One area of ongoing debate and investigation is the role of salvage ASCT in MM. Salvage ASCT involves the use of previously collected and cryopreserved PBSCs for a second transplant in cases of disease relapse or progression [6,7]. Published data show that while salvage ASCT can be effective, its utilization has been declining in recent years [8]. This decline can be attributed to several factors, including the advent of novel therapeutic agents and concerns about the cost-effectiveness of storing and utilizing stored stem cells [7,8].

High-dose chemotherapy (HD-CHT) followed by autologous stem cell transplantation (ASCT) has long been established as the standard of care for eligible patients with multiple myeloma (MM), remaining relevant even in the era of innovative therapeutic approaches [9]. Both IFM 2009 and the DETERMINATION study confirmed a longer PFS in the upfront transplant group compared to non-transplant path. ASCT has demonstrated improved outcomes in MM patients, with clinical trials suggesting its efficacy as salvage therapy after prolonged remission [10]. The primary source of hematopoietic stem cells for ASCT has shifted toward peripheral blood stem cells (PBSCs). The challenges associated with collecting additional PBSC grafts post-initial myeloablative conditioning have led many transplant centers to adopt a practice of preemptively collecting and storing adequate PBSCs during first-line therapy [11]. This strategy accounts for the potential need for tandem transplants or salvage treatments, given the intricacies of mobilization during relapse.

### 1.4. Cost-Effectiveness and Resource Utilization

A crucial aspect of stem cell collection policy in MM revolves around cost-effectiveness and resource utilization.

However, recent developments in MM treatment modalities, including highly effective combination therapies, bispecific antibodies, and cellular therapies, raise questions about the rationale and value of salvage ASCT for relapsed/refractory MM (RRMM). Despite the availability of stored PBSC grafts, salvage ASCT is not always performed due to various factors such as deterioration in performance [12], status, or therapy-related comorbidities. Furthermore, a cost utilization analysis conducted in 2013 revealed that a substantial proportion of patients had PBSC products remaining unused in prolonged cryopreservation after the initial ASCT [3,13]. This surplus stemmed from the estimated cost of collecting and storing PBSCs beyond that required for a single ASCT, accounting for approximately one-third of total costs.

The average cost for PBSC collection, cryopreservation, and storage was reported to be at least $20,065.96 per person, with an additional $6718.11 per person spent on collecting and storing PBSCs for a second ASCT that often went unrealized [8,14]. Another analysis confirmed this trend, with less than 10% of patients ultimately utilizing their stored PBSC grafts over a ten-year period [15].

These findings beg the crucial question: do we still need backup stem cells for MM patients, and if so, for which patients is this backup likely to be beneficial?

## 2. Methods

A local institutional review board (IRB)-approved, single-center, retrospective study was conducted on all patients 18 years or older who received ASCT for a diagnosis of MM from 1 January 2012 to 23 June 2022. Patients were included if they were aged 18 years or older and received their first ASCT for a diagnosis of MM. Patients who received a single ASCT and received all the collected stem cells with no cells remaining in storage were excluded from our analysis.

Liquid nitrogen was used to cryopreserve stem cells for autologous stem cell transplants because cryopreservation media are thermally unstable at higher storage temperatures. Stem cells are preserved in liquid nitrogen at temperatures ranging from −145 to −196 °C. This method allows for long-term storage of stem cells for years.

In order to tackle this pertinent query and alleviate the economic strain entailed in the collection, processing, and storage of peripheral blood stem cells (PBSCs), we embarked on a comprehensive investigation. Our research aimed to pinpoint a specific subgroup of multiple myeloma (MM) patients who were less inclined to undergo salvage ASCT, thereby obviating the need to preserve excess stem cells. Our data analysis encompassed the entire cohort of MM patients who underwent their initial ASCT procedure from January 2012 to June 2022. We carefully excluded individuals who had originally planned for tandem transplants and those who had undergone a single ASCT without any remaining stored stem cells. It is important to note that our analytical endeavors were conducted exclusively within the confines of a single, specialized stem cell transplant center.

## 3. Results

We present here an analysis of the descriptive statistics derived from a cohort of eligible multiple myeloma (MM) patients, totaling 240 individuals who successfully collected an adequate quantity of peripheral blood stem cells (PBSCs) for two autologous stem cell transplantations (ASCTs). The median age of these patients at the time of PBSC collection was 61 years, with an age range spanning from 32 to 77. Our data closely align with the previously published literature, providing insights into the evolving landscape of ASCT utilization in MM treatment.

We categorized our patient cohort based on gender, age groups, racial background, and the average number of days required for PBSC collection. Each patient received a cell dose of 10 × 10^6^ CD34+ cells per kilogram of body weight. Notably, our cohort consisted of more males than females, with 132 male patients and 108 female patients. Furthermore, we observed a higher prevalence of African American patients, comprising 59% of the cohort (Table 1). 

A notable trend that emerged from our analysis is the declining utilization of salvage ASCT within our cohort, with only a mere 7% of patients, specifically 17 out of the 240 patients, opting for this approach. Further examination of the data revealed intriguing patterns regarding the age distribution of patients who underwent salvage ASCT. Particularly striking was the observation that salvage ASCT was more common among younger patients; the majority of patients who received both salvage ASCT were 61 years old or younger at the time of their first ASCT.

Descriptive survival data indicates a numerical difference in median survival between the two groups. Patients who underwent a single ASCT had a median survival of 1.9 years, whereas those who underwent two ASCTs had a longer median survival of 4.6 years. The median follow-up period was the same as the median survival time for each group, reflecting consistent monitoring durations.

**Figure 1:**
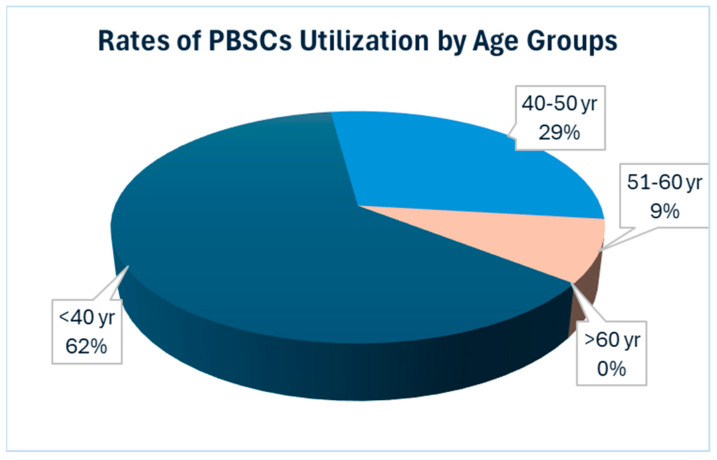
Showing the rates of utilization of stored PBSCs according to age group. The data reveals a higher utilization rate among younger patients, with the highest rate observed in the age group under 40 years. Conversely, there is no utilization of stored PBSCs for salvage ASCT in patients over 60 years old. Abbreviation: PBSCs = Peripheral blood stem cells.

Conversely, our data underscored a lesser utilization of salvage ASCT in the elderly population, defined here as individuals aged over 60 years. Additionally, we observed a greater proportion of patients in the younger age groups opting for salvage ASCT. Among the patients under 50 years old, six out of 30 underwent salvage ASCT, while three out of seven patients under 40 years old chose this option, accounting for 20% and 43%, respectively. These findings underscore the relatively lower utilization of salvage ASCT among the elderly population, suggesting that considering PBSC collection for a single transplant may be a more cost-effective strategy (Figure 1).

This observation has prompted us to propose a potentially cost-effective strategy of focusing PBSC collection efforts on a single transplant among elderly MM patients.

Additionally, many stem cell transplantation centers traditionally adopt a strategy of collecting PBSCs at a target of 10 × 10^6^ CD34+ cells per kilogram and then splitting the collected PBSCs into two. Furthermore, it has been a common practice to store all the collected PBSCs, even if they surpass the collection target. Among the patients who completed PBSC collection in a single day, 127 out of 240 had an average of four bags per patient.

Remarkably, 97 patients collected more than 10 × 10^6^ CD34+ cells per kilogram, resulting in a total of 395 stored bags. The optimal number of bags for these 97 patients should have been 194. By revising our practice and discarding any excess PBSCs, we could potentially reduce our stored PBSCs by half, leading to substantial cost savings in terms of processing, storage, and space requirements.

Our analysis revealed that patients requiring more than one day for PBSC collection to meet this threshold were typically aged 65 years or older. By reorienting our practice to emphasize PBSC collection for a single transplant among elderly patients, we could potentially reduce the number of collection days and associated costs, including those related to collection procedures, processing, storage, staffing, and storage space requirements.

In addition to optimizing PBSC collection strategies, we advocate for the development of proactive approaches for patients who exceed their collection goals on the first day of leukapheresis. We propose implementing strategies for the responsible disposal or repurposing of excess PBSCs, particularly for valuable stem cell research purposes. Crucially, this decision-making process should be initiated on the collection day itself to circumvent the added financial burden associated with storing and maintaining surplus products.

## 4. Discussion

Published data on the topic of stem cell collection for autologous stem cell transplantation (ASCT) in multiple myeloma (MM) patients has provided key insights into the evolving landscape of MM treatment and the financial implications of stem cell storage. The following key findings have emerged from the published literature in addition to our data (Table 2).

Efficacy of ASCT: clinical trials have consistently demonstrated the improved outcomes associated with ASCT in MM patients, even highlighting the potential effectiveness of salvage ASCT following prolonged remission [11].

Stem Cell Processing: A cost utilization analysis conducted in 2013 revealed that a substantial proportion (approximately 70%) of MM patients had PBSC products that remained unused and cryopreserved after the initial ASCT. The cost of collecting and storing excess PBSCs beyond what was necessary for a single ASCT accounted for a significant portion of total costs [14].

Financial Burden: the estimated average cost for PBSC collection, cryopreservation, and storage was at least $20,065.96 per person, with additional expenses of at least $6718.11 per person spent on collecting and storing PBSCs for a second ASCT that often went unrealized [12].

Declining Utilization of Salvage ASCT: a more recent analysis from 2016 corroborated the trend of decreasing utilization of stored PBSC grafts, with less than 10% of patients ultimately using their stored cells over a ten-year period.

Novel Myeloma Targeting Agents: The recent approval and integration of novel myeloma-targeting agents such as CAR T-cell therapies and bispecific antibodies have significantly impacted the use of salvage autologous stem cell transplantation (ASCT) in multiple myeloma (MM) treatment. These innovative therapies offer new treatment options that can potentially replace the need for second transplants, particularly in patients with relapsed or refractory MM. However, the cost of these new therapeutics is substantial. CAR T-cell therapies, for instance, can cost hundreds of thousands of dollars per treatment, and bispecific antibodies also come with high price tags, and health care burden. These costs can place a significant financial burden on healthcare systems and patients. Therefore, while these novel therapies offer promising alternatives to traditional treatments, a cautious and judicious approach is necessary when integrating them into clinical practice. Careful patient selection and case-by-case cost–benefit analyses are essential to ensure that the benefits of these expensive treatments justify their high costs.

Age-Based Utilization: The therapeutic landscape for multiple myeloma (MM), particularly in elderly patients, has evolved significantly, with current guidelines emphasizing tailored treatment approaches based on patient age, performance status, and comorbidities. The International Myeloma Working Group (IMWG) has provided specific recommendations for the management of MM in elderly patients, highlighting the importance of balancing efficacy and tolerability.

In elderly MM patients, frontline treatment often includes less-intensive regimens compared to younger patients. Common approaches involve combinations of immunomodulatory drugs (IMiDs) like lenalidomide, proteasome inhibitors such as bortezomib, and corticosteroids. These regimens aim to achieve optimal disease control while minimizing adverse effects. However, the role of high-dose chemotherapy (HD-CHT) followed by autologous stem cell transplantation (ASCT) is nuanced in this population.

The eligibility for ASCT in elderly patients is generally determined by biological age, performance status, and comorbidities rather than chronological age alone. Guidelines suggest that fit elderly patients, often those below 70–75 years without significant comorbid conditions, may still benefit from ASCT. However, the utilization of salvage ASCT in elderly patients remains limited due to factors such as decreased performance status and increased therapy-related toxicity.

Given these guidelines, our proposed strategy for PBSC collection, particularly targeting single-transplant collections for elderly MM patients, aligns with current therapeutic approaches. By focusing on a single PBSC collection for this population, we aim to optimize resource utilization and reduce unnecessary costs associated with the collection, processing, and storage of excess PBSCs. This approach not only supports cost-effectiveness but also aligns with the evolving treatment paradigms that prioritize tailored, patient-centric care in the management of elderly MM patients.

Implementing such strategies requires careful patient selection and adherence to guidelines to ensure that the benefits of ASCT, when applicable, are maximized, while minimizing the potential risks and financial burdens associated with excessive stem cell collection and storage.

While our study, in collation with the published literature, provides valuable insights into the utilization patterns of ASCT and PBSC collection strategies, the study has several limitations which highlight the need for further research. The nature of this study is primarily descriptive. As a result, it provides a broad overview of trends and observations but lacks the depth of analysis required to establish causal relationships. We provided this descriptive insight to help identify real-world patterns and generate hypotheses. However, this study does not account for potential confounding variables or underlying mechanisms driving the observed outcomes.

Future studies should incorporate more detailed clinical and treatment-related variables to better understand the factors influencing ASCT outcomes and optimize stem cell collection and utilization practices in multiple myeloma patients.

In summary, while ASCT remains a crucial therapy for MM, there is an evolving landscape regarding the utilization of stem cell collection, with a growing emphasis on cost-effective strategies and responsible management of excess PBSCs. These findings from the published literature provide valuable insights into optimizing ASCT protocols and reducing the financial burden associated with stem cell collection and storage.

## 5. Conclusions

Our findings emphasize the potential benefits of an age-based approach to stem cell collection, particularly targeting elderly MM patients for PBSC collections tailored to a single transplant. This approach could significantly reduce the financial burden associated with excess stem cell collection and storage, while ensuring that resources are efficiently allocated to patients most likely to benefit.

Furthermore, the proposed strategies for the disposal or repurposing of excess PBSCs on the collection day align with prudent resource management. By implementing these measures, we can potentially reduce costs associated with storing unused PBSCs.

In summary, the utilization of salvage ASCT in MM patients is decreasing, prompting the need for revised policies to adapt to the changing treatment landscape. We advocate for considering elderly MM patients for PBSC collections tailored to a single transplant, implementing efficient disposal strategies for excess PBSCs, and making these decisions on the collection day. These measures aim to strike a balance between optimizing patient care and resource utilization in the era of evolving MM therapeutics.

## Figures and Tables

**Table 1:** Baseline demographics.

	Transplanted MM Patients Who Collected Sufficient Cell Dose for 2 ASCT	
	Patients Underwent Single ASCT (*n* = 223)	Patients Underwent 2 ASCT (*n* = 17)
Gender		
Male	121 (54%)	11 (65%)
Female	102 (46%)	6 (35%)
Race		
Caucasian/White	89 (40%)	8 (47%)
African American/Black	133 (60%)	8 (47%)
Other	1 (0%)	1 (6%)
Age at First ASCT		
30–39	4 (2%)	3 (18%)
40–49	20 (9%)	3 (18%)
50–59	70 (31%)	6 (34%)
60–69	93 (42%)	3 (18%)
70–79	36 (16%)	2 (12%)
Collection		
Average Number of Collections	1.01	1
Average Number of Days of Collection	1.56	1.76
Average CD34+ Cell Count Collected (×10^6^/kg)	11.62	11.3
Median CD34+ Cell Count Collected (×10^6^/kg)	10.93	9.12
Survival		
Median Survival (years)	1.9	4.6
Median Follow Up (years)	1.9	4.6

Abbreviations: MM = Multiple Myeloma; ASCT = Autologous Stem Cell Transplantation.

**Table 2:** Key Findings of Utilization of Stem Cell Collection.

Observed Factors	Description
Standard of Care	HD-CHT and ASCT are the standard of care for eligible MM patients.
Improved Outcomes with ASCT	Clinical trials have shown improved outcomes with ASCT, including potential use in salvage ASCT.
PBSCs as the Primary Source	PBSCs have become the primary source of hematopoietic stem cells for ASCT in MM.
Collection Challenges	Collecting additional PBSC grafts after initial conditioning can be challenging, leading to excess collection.
Changing Landscape	The influx of novel treatments may reduce the need for salvage ASCT, raising questions about its value.
Underutilization of Stored PBSCs	Despite storage, salvage ASCTs are not always performed due to various reasons, contributing to underutilization.
Financial Burden	Cost analyses highlight a financial burden associated with excess stem cell collection and storage.
Strategies for Cost Reduction	Identifying patients less likely to need salvage ASCT can reduce the need for excess PBSC storage.
Age-Based Strategy	Age is a relevant factor, with younger patients more likely to undergo salvage ASCT.
Excess PBSC Disposal	Strategies for responsible disposal or repurposing of excess PBSCs can reduce storage costs.

This table provides a concise overview of the main findings and key considerations regarding stem cell collection in the context of ASCT for MM. Abbreviations: HD-CHT = High-dose chemotherapy; ASCT = Autologous Stem Cell Transplantation; MM = Multiple Myeloma; PBSC = Peripheral blood stem cell.

## Data Availability

Data used in the study is unavailable due to privacy and ethical restriction of the institution.

## References

[1] Yang Y., Li J., Wang W., Wang Y., Maihemaiti A., Ren L., Lan T., Zhou C., Li P., Wang P. (2023). The evolving diagnosis and treatment paradigms of multiple myeloma in China: 15 years’ experience of 1256 patients in a national medical center. Cancer Med..

[2] Gandolfi S., Vekstein C., Laubach J.P., O’Brien A., Masone K., Munshi N.C., Anderson K.C., Richardson P.G. (2018). The Evolving Role of Transplantation in Multiple Myeloma: The Need for a Heterogeneous Approach to a Heterogeneous Disease. Clin. Adv. Hematol. Oncol..

[3] Sauer S., Hieke L., Brandt J., Müller-Tidow C., Schmitt A., Kauer J., Kriegsmann K. (2023). Impact of Clinical Parameters and Induction Regimens on Peripheral Blood Stem-Cell Mobilization and Collection in Multiple Myeloma Patients. Transfus. Med. Hemotherapy.

[4] Kriegsmann K., Wuchter P. (2023). Stem Cell Mobilization, Collection, and Processing. Transfus. Med. Hemother..

[5] Ji M.M., Shen Y.G., Gong J.C., Tang W., Xu X.Q., Zheng Z., Chen S.Y., He Y., Zheng X., Zhao L.D. (2023). Efficiency and safety analysis of Plerixafor combined with granulocyte colony-stimulating factor on autologous hematopoietic stem cell mobilization in lymphoma. Zhonghua Xue Ye Xue Za Zhi.

[6] Khan A.M., Ozga M., Bhatt H., Faisal M.S., Ansari S., Zhao Q., Bumma N., Cottini F., Devarakonda S., Rosko A. (2023). Outcomes After Salvage Autologous Hematopoietic Cell Transplant for Patients with Relapsed/Refractory Multiple Myeloma: A Single-Institution Experience. Clin. Lymphoma Myeloma Leuk..

[7] Khan S., Reece D., Atenafu E.G., Bhella S., Chen C., Masih-Khan E., Paul H., Prica A., Tiedemann R., Trudel S. (2023). Post Salvage Therapy Autologous Transplant for Relapsed Myeloma, Ongoing Relevance within Modern Treatment Paradigms?. Clin. Lymphoma Myeloma Leuk..

[8] Wolf J., Smythe J., Griffin J. (2021). Letter to the Editor Regarding “Utilization and Cost Implications of Hematopoietic Progenitor Cells Stored for a Future Salvage Autologous Transplantation or Stem Cell Boost in Myeloma Patients”. Transplant. Cell Ther..

[9] Goldsmith S.R., Vij R. (2021). Evolving Paradigms of Therapy for Multiple Myeloma: State of the Art and Future Directions. JCO Oncol. Pract..

[10] Voorhees P.M., Usmani S.Z. (2016). The role of high-dose melphalan and autologous stem cell transplant in the rapidly evolving era of modern multiple myeloma therapy. Clin. Adv. Hematol. Oncol..

[11] Wei X., Wei Y. (2023). Stem cell mobilization in multiple myeloma: Challenges, strategies, and current developments. Ann. Hematol..

[12] Krummradt F., Sauer S., Pavel P., Klein E.-M., Schmitt A., Kriegsmann M., Jordan K., Müller-Tidow C., Goldschmidt H., Wuchter P. (2020). Storage, Utilization, and Disposal of Hematopoietic Stem Cell Products in Patients with Multiple Myeloma. Biol. Blood Marrow Transplant..

[13] Kriegsmann K., Bittrich M., Sauer S., Tietze-Stolley C., Movassaghi K., Grube M., Vucinic V., Wehler D., Burchert A., Schmidt-Hieber M. (2023). Mobilization and Hematopoietic Stem Cell Collection in Poor Mobilizing Patients with Lymphoma: Final Results of the German OPTIMOB Study. Transfus. Med. Hemotherapy.

[14] Chhabra S., Thapa B., Szabo A., Konings S., D’Souza A., Dhakal B., Jerkins J.H., Pasquini M.C., Johnson B.D., Hari P.N. (2020). Utilization and Cost Implications of Hematopoietic Progenitor Cells Stored for a Future Salvage Autologous Transplantation or Stem Cell Boost in Myeloma Patients. Biol. Blood Marrow Transplant..

[15] Gertz M.A., Wolf R.C., Micallef I.N.M., Gastineau D.A. (2010). Clinical impact and resource utilization after stem cell mobilization failure in patients with multiple myeloma and lymphoma. Bone Marrow Transplant..

